# Antioxidant Therapies in Traumatic Brain Injury

**DOI:** 10.3390/antiox9030260

**Published:** 2020-03-22

**Authors:** Valentina Di Pietro, Kamal M. Yakoub, Giuseppe Caruso, Giacomo Lazzarino, Stefano Signoretti, Aron K. Barbey, Barbara Tavazzi, Giuseppe Lazzarino, Antonio Belli, Angela Maria Amorini

**Affiliations:** 1 Neurotrauma and Ophthalmology Research Group, Institute of Inflammation and Aging, University of Birmingham, Birmingham B15 2TT, UK; V.DiPietro@bham.ac.uk (V.D.P.); k.yakoub@bham.ac.uk (K.M.Y.); 2 NIHR Surgical Reconstruction and Microbiology Research Centre, University Hospitals Birmingham NHS Foundation Trust, Birmingham B15 2TT, UK; 3 The Beckman Institute for Advanced Science and Technology, University of Illinois at Urbana Champaign, Champaign, IL 61801, USA; barbey@illinois.edu; 4 Department of Laboratories, Oasi Research Institute – IRCCS, Via Conte Ruggero 73, 94018 Troina (EN), Italy; forgiuseppecaruso@gmail.com; 5 UniCamillus, Saint Camillus International University of Health Sciences, Via di Sant’Alessandro 8, 00131 Rome, Italy; giacomo.lazzarino@unicamillus.org; 6 UOC Neurochirurgia, ASL Roma2, S. Eugenio Hospital, Piazzale dell’Umanesimo 10, 00144 Rome, Italy; stefano.signoretti@aslroma2.it; 7 Institute of Biochemistry and Clinical Biochemistry, Catholic University of Rome, Largo F.Vito 1, 00168 Rome, Italy; 8 Department of Scienze di laboratorio e infettivologiche, Fondazione Policlinico Universitario A. Gemelli IRCCS, Largo A. Gemelli 8, 00168 Rome, Italy; 9 Department of Biomedical and Biotechnological Sciences, Division of Medical Biochemistry, University of Catania, Via S.Sofia 97, 95123 Catania, Italy; amorini@unict.it

**Keywords:** traumatic brain injury, oxidative/nitrosative stress, low molecular weight antioxidants, concussion

## Abstract

Due to a multiplicity of causes provoking traumatic brain injury (TBI), TBI is a highly heterogeneous pathology, characterized by high mortality and disability rates. TBI is an acute neurodegenerative event, potentially and unpredictably evolving into sub-chronic and chronic neurodegenerative events, with transient or permanent neurologic, cognitive, and motor deficits, for which no valid standardized therapies are available. A vast body of literature demonstrates that TBI-induced oxidative/nitrosative stress is involved in the development of both acute and chronic neurodegenerative disorders. Cellular defenses against this phenomenon are largely dependent on low molecular weight antioxidants, most of which are consumed with diet or as nutraceutical supplements. A large number of studies have evaluated the efficacy of antioxidant administration to decrease TBI-associated damage in various animal TBI models and in a limited number of clinical trials. Points of weakness of preclinical studies are represented by the large variability in the TBI model adopted, in the antioxidant tested, in the timing, dosages, and routes of administration used, and in the variety of molecular and/or neurocognitive parameters evaluated. The analysis of the very few clinical studies does not allow strong conclusions to be drawn on the real effectiveness of antioxidant administration to TBI patients. Standardizing TBI models and different experimental conditions, as well as testing the efficacy of administration of a cocktail of antioxidants rather than only one, should be mandatory. According to some promising clinical results, it appears that sports-related concussion is probably the best type of TBI to test the benefits of antioxidant administration.

## 1. Introduction

Traumatic brain injury (TBI) affects more than 10 million people worldwide each year, representing 30% to 40% of all injury-related mortalities and disabilities among all age groups, with enormous social and economic impacts [1,2]. Epidemiological previsions until 2030 indicate a 2–3-times higher incidence of patients suffering from TBI-related disabilities than those with neurological disabilities from Alzheimer’s disease or cerebrovascular disorders. Despite recent advances in trauma research and the ongoing efforts of collaborative multidisciplinary studies to tackle this problem and improve patients’ outcomes, TBI still represents a major global health burden and public health challenge among all ages in all countries regardless of the patient’s income level.

### 1.1. Definition/Classification

TBI results from the absorption by the cerebral tissue of part of the energy associated with an external mechanical force, not necessarily acting directly to the head. This amount of energy causes the derangement of a myriad of biochemical, metabolic, and molecular functions, deeply affecting brain cell homeostasis and leading to temporary or permanent impairment of consciousness, neurocognitive deficits, neuromotor disabilities, or psychological disturbances [3,4]. The extent to which these mechanisms are damaged in TBI depends on the severity of the impact. There are various systems and scales to assess the severity of TBI, the most commonly used is the Glasgow Coma Scale (GCS) which classifies TBI into mild (GCS range 13–15), moderate (GCS range 9–12) and severe (GCS range 3–8). The GCS is obtained by scoring specific clinical assessments, including eye opening, motor and verbal responses [5] (Table 1). 

**Figure 1:**
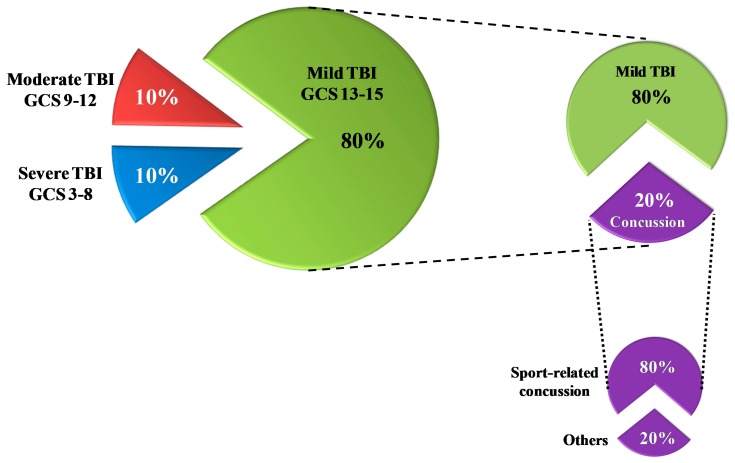
Rates of traumatic brain injury (TBI) according to the severity classification based on the Glasgow Coma Scale (GCS) score. Of the total TBI, 80% are mild TBI. Of these, 20% are concussions; 80% of all concussions are sports-related concussions.

As shown in Figure 1, the majority of TBI patients (80%) sustain mild injuries [6], which have less mortality rates compared to both moderate and severe injuries (30–40%) [7].

**Figure 2:**
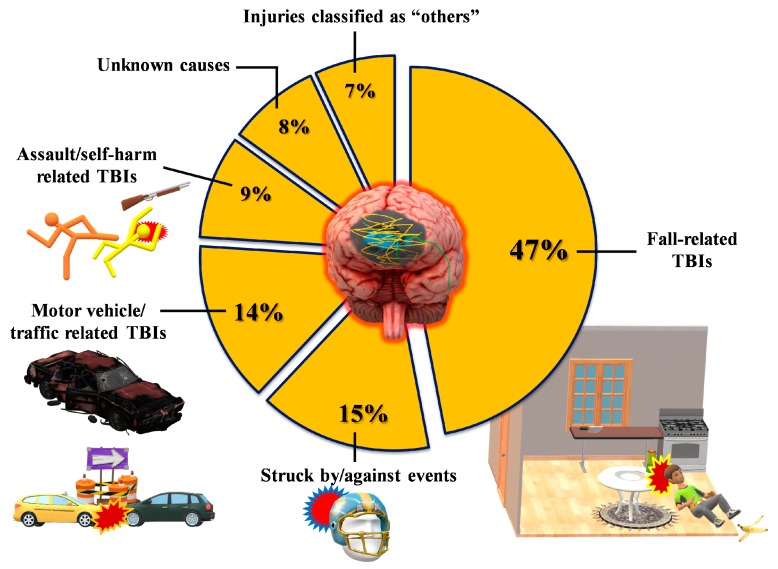
Classification of TBI patients in terms of rates and types of the most frequent events causing a traumatic head injury. Data refer to USA epidemiological data, which are currently the most accurate worldwide.

According to the available epidemiological data [1,6,8], men are more prone to sustain TBI, which is caused, more than 50% of the time, by falls and road traffic accidents (Figure 2). 

Unfortunately, an increasing number of severe TBIs occur during brawls, and this is often due to gunshots, with very devastating consequences. 

### 1.2. Triage

After assessing the injury severity, pre-hospital management at the scene of TBI occurrence is mainly conducted to maintain vital physiological functions of the patient (airway, breathing, circulation, spinal stability) until their transfer to an emergency department [9,10]. In the emergency department, clinicians assess the sustained injuries and continue the supportive treatment, often performing conventional neuroradiological exams to scan the head (and cervical spine on occurrence) by using computed tomography (CT) and/or magnetic resonance imaging (MRI). These initial interventions depend on the severity of TBI and the national/local guidelines. Neurosurgeons assess the surgical need and establish the treatment approach and management plan [9,10]. 

### 1.3. Therapy

Due to the heterogeneity of TBI and limited knowledge of the underlying pathophysiological mechanisms, there are no current standardized surgical and pharmacological treatments for TBI patients. Despite their promising preclinical outcomes, many intervention strategies have failed to demonstrate beneficial effects in randomized controlled trials so that TBI patients are still waiting for the discovery of drugs capable of decreasing mortality and disability associated with this pathology [11]. The primary insult resulting from the application of mechanical force in TBI occurs immediately and is inevitable. The amount of permanent damage due to sudden neuronal death and permanent loss of cerebral tissue functions is also inevitable, so that all the efforts at this stage are to prevent the primary injury from happening [12]. The secondary insult, involving biochemical and molecular processes described in the next paragraph, may last for days, weeks, or months, and its damaging activity might actively be decreased by proper therapies [12]. This secondary insult is characterized by the release of excitatory neurotransmitters (glutamate, aspartate), glucose dysmetabolism with mitochondrial dysfunction, and free radical overproduction. Final consequences are the activation of different molecular pathways and inflammatory cascades, leading to cellular apoptosis and damage of the blood–brain barrier (BBB) permeability [12].

As previously stated, treatments deeply vary because of the heterogeneity of TBI and the patient’s response to therapy and it is almost impossible to establish standard approaches to be used in all TBI patients. At present, the drugs most frequently administered are aimed at controlling intra-cranial pressure (ICP) within normal levels (<22 mm Hg) [13], maintaining cerebral blood flow and decreasing secondary injury associated damage [12]. A list of these drugs is summarized in Table 2 [10,11,12,13,14].

Acute neurosurgical intervention is often required in moderate to severe TBI patients to evacuate hematoma, causing compression of the brain within the closed skull and leading to the rise of intracranial pressure (ICP). TBI patients needing surgical intervention should be managed in centers with appropriate neurosurgery staff and a neurosurgical intensive care unit (NICU) [10]. A frequent medical intervention is aimed at avoiding the ICP raised by controlling ventilation in order to reduce partial CO_2_ pressure, a potent vasodilator. In the case of brain edema, the intravenous infusion of hyperosmolar agents is a common clinical practice targeted to improve the blood rheology and cerebral blood flow (CBF) [10,12]. Moderate to severe TBI patients often undergo therapy to induce pharmacological paralysis and sedation aimed at avoiding an increase in ICP and to reduce the metabolic energy requirements of the brain [10,12]. It is highly recommended that mildly injured TBI patients, particularly those who score <15 on the GCS, even in the absence of gross clinical symptoms, are admitted for neurological observation and conventional neuroradiological evaluation (CT scan, MRI), in order to exclude the presence of subdural and/or subarachnoid hematomas [10].

## 2. TBI and Oxidative/Nitrosative Stress: A Rationale for Antioxidant-Based Therapies

**Figure 3:**
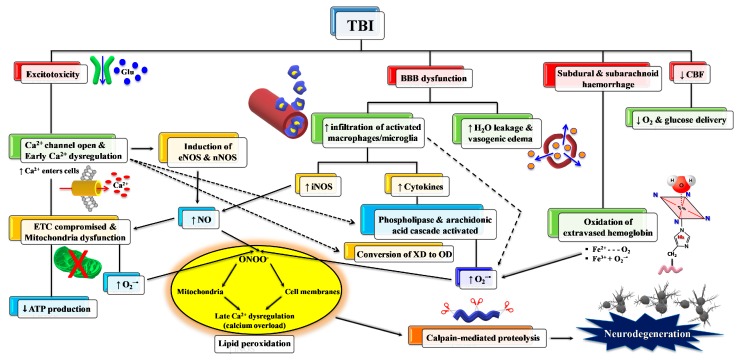
Schematic representation of some of the main pathological processes characterizing the TBI-associated secondary insult. The force discharged and partly absorbed by the cerebral tissue at the time of impact (primary insult) induces immediate glutamate release by neurons, change in the blood–brain barrier (BBB) permeability, frequent hemorrhage, and decrease in the cerebral blood flow (CBF). Excitotoxic phenomena due to sustained glutamate (Glu) release deeply alter ionic homeostasis, particularly causing an increase in mitochondrial Ca^2+^. Malfunctioning of the mitochondrial electron transport chain (ETC) and oxidative phosphorylation (OXPHOS) is consequent to increased Ca^2+^ entry and decreased oxygen and glucose delivery (due to a decrease in CBF), and ultimately generating decreased ATP formation with an energy crisis. Ca^2+^ also activates endothelial (eNOS) and neuronal (nNOS) isoforms of nitric oxide (NO) synthase, promotes the conversion of xanthine dehydrogenase (XDH) into xanthine oxidase (XO), and triggers the arachidonic acid cascade activating phospholipases. The change in BBB permeability modifies water vascular permeability, causing vasogenic brain edema, and allows infiltration and activation of macrophages/microglia, that are responsible either for NO overproduction by the inducible NO synthase (iNOS) or for the release of pro-inflammatory cytokines. Hematomas generate the release of hemoglobin (Hb) from ruptured erythrocytes and the consequent oxidation of Fe^2+^ of Hb to Fe^3+^. This last process, together with XO activity, the arachidonic acid cascade, activated macrophages/microglia, and dysfunctional mitochondria, generates a flow of superoxide anion (O_2_•^−^) and gives rise to the reaction with NO and the formation of peroxynitrite (ONOO•^−^). The damaging action of ROS and RNS on polyunsaturated fatty acids of phospholipids of biological membranes triggers a lipid peroxidation reaction chain, culminating in neuronal cell death.

As mentioned above, primary TBI injury is the damage occurring to the tissue (cerebral cells, blood vessels) when part of the energy, associated with the mechanical force causing the injury, is discharged against nerve and blood vessel cells. Secondary TBI injury refers to a cascade of biochemical and molecular mechanisms triggered by the primary insult. These neurochemical changes start immediately after impact, last for hours, days, or weeks depending on the injury severity, and may culminate in cerebral cell death with a loss of neuronal functions. The secondary insult is characterized by the imbalance of ionic homeostasis, release of excitatory neurotransmitters (glutamate, aspartate), glucose dysmetabolism with mitochondrial dysfunction, and free radical overproduction. Final consequences are the activation of different molecular pathways and inflammatory cascades, leading to cellular apoptosis and damage of the BBB permeability [15]. The formation of reactive oxygen species (ROS) and free radicals in brain tissue following TBI is well documented and plays a crucial role in triggering molecular damaging processes (lipid peroxidation, DNA damage, protein oxidation) and in exacerbating glutamate excitotoxicity, mitochondrial dysfunction, ionic dysregulation, and activation of cellular proteases [16,17,18]. A schematic representation of secondary injury is illustrated in Figure 3.

**Figure 4:**
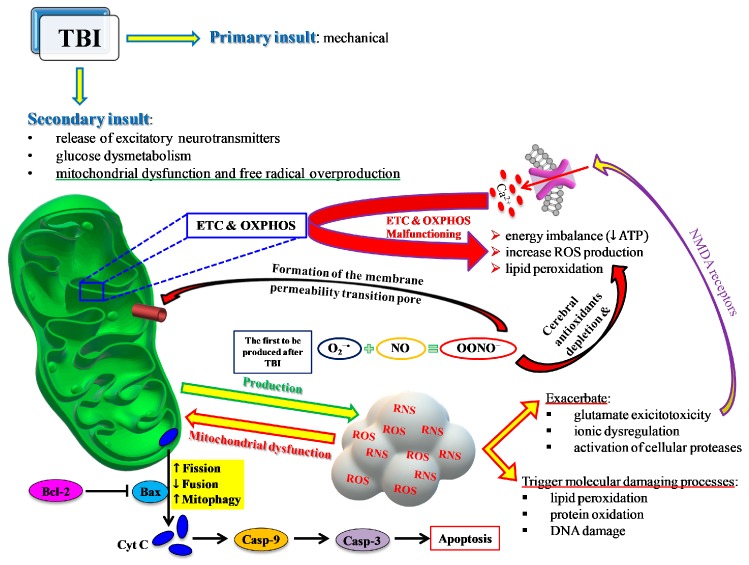
Schematic representation of the central role played by mitochondria in most of the events characterizing the TBI-mediated secondary insult. ETC and OXPHOS are impaired by the increase in Ca2+ and the change in the mitochondrial quality control (MCQ) network. A decrease in fusion (OPA1, MFN1, MFN2) and an increase in fission (DRP1, FIS1) and mitophagy (PINK1, PARK2) greatly contribute to the reduced mitochondrial phosphorylating capacity, unbalanced ATP production and consumption, leading to an energy crisis. Concomitantly, an insurgence of oxidative/nitrosative stress takes place due to the overproduction of ROS and RNS exceeding the cell antioxidant defenses. The intrinsic pathway of apoptosis via cytochrome c release and caspase activation leads to increasing cerebral cell death.

Glutamate release is one of the main processes activated after TBI [19] that causes an influx of Ca^2+^ into neuronal cells via activation of NMDA receptors [20] and leads to an “early” calcium dysregulation. This negatively impacts the main mitochondrial function, namely, the electron transport chain (ETC) coupled with oxidative phosphorylation (OXPHOS) for energy production (ATP), thus leading to energy imbalance [21,22] and contributing to increased ROS production [23] with documented post-traumatic membrane lipid peroxidation [24] (Figure 4). 

Dysfunctional mitochondria are also characterized by an imbalance of the mitochondrial quality control network, regulating fusion, fission, and mitophagy. These processes, also known as mitochondrial dynamics, are tightly controlled by a set of proteins selectively promoting fusion (optic dominant atrophy 1, OPA1; mitofusin 1 and 2, MFN1 and MFN2), fission (mitochondrial fission protein 1, FIS1; dynamin-related protein 1, DRP1) or mitophagy (mitochondrial serine/threonine-protein kinase, PINK1; parkin, PARK2). Moderate to severe TBI inhibit fusion and activate fission and mitophagy, consequently decreasing both the total number of mitochondria and the number of those properly functioning. In this light, it has been shown that the peptide SS-31, specifically targeting the mitochondrial phospholipid cardiolipin and providing significant neuroprotection in a variety of neurological diseases, plays a significant role as a potential agent to reduce TBI-mediated mitochondrial dysfunction and oxidative/nitrosative stress [25]. Its pre-impact administration decreased ROS-mediated damage, inhibited apoptosis, and improved mitochondrial biogenesis, thus providing significant neuroprotection in experimental TBI in mice [25].

Among ROS, superoxide anion is the first to be produced after TBI by cerebral cells via different mechanisms, such as the activation of phospholipases and the arachidonic acid cascade (cycloxygenase, COX), the conversion of xanthine dehydrogenase to xanthine oxidase, and, mainly, the malfunctioning of mitochondrial ETC [26]. Activated microglia and infiltrating neutrophils and macrophages also provide additional sources of superoxide radical [27,28] at later time points after TBI, very frequently through the activity of NADPH-oxidase (NOX) [29]. Oxidation of Fe^2+^ of extravasated hemoglobin (Hb), as a result of the rupture of cerebral blood vessels, may also provide a further source of ROS. Physiologically, superoxide anion is rapidly and efficiently converted into H_2_O_2_ + O_2_ by the enzyme superoxide dismutase (SOD) [29], and H_2_O_2_ is then detoxified into O_2_ + H_2_O mainly by glutathione peroxidase and, partly, by catalase and peroxiredoxins. Superoxide anion overproduction by the aforementioned mechanisms, coupled with acidosis frequently occurring after TBI, favor H_2_O_2_ to react with reduced iron of extravasated Hb, producing hydroxyl radicals through the Fenton reaction. Hydroxyl radicals have no specific defense antioxidants, have a tremendously high oxido-reductive potential, and can easily attack nearly any biological molecule capable of donating one electron and causing its irreversible modification [26]. In addition, oxidized Fe^3+^ (either from extravasated Hb or released from ferritin) contributes to the hydroxyl radical generation through the reaction of Fe^3+^ with superoxide anions (the Haber–Weiss reaction) which regenerates Fe^2+^ for further Fenton reactions. The high oxido-reductive potential allows hydroxyl radicals to tear off one H• from double bonds of polyunsaturated membrane phospholipids, thus initiating the dangerous lipid peroxidation reaction chain disrupting the functions and integrities of biological membranes [30]. 

TBI also greatly affects the homeostasis of nitric oxide (NO). NO is a fundamental signaling gaseous molecule in the nervous, immune, and cardiovascular systems, transmitting both intracellular and intercellular signals crucial for cell and organ survival. It is produced by the activity of a family of enzymes named nitric oxide synthases (NOS). The 3 NOS isoforms (endothelial, neuronal, and inducible) are not equally upregulated after TBI [31,32,33]. Depending on which isoform is more upregulated, NO may or may not exert beneficial effects. For example, although increased NO synthesis occurs early after TBI, the amount generated by nNOS and eNOS [33,34] has a crucial role as a vasodilator in maintaining cerebral blood flow [35]. In contrast, NO formed intracellularly in cerebral cells by iNOS is deeply involved in the generation of reactive nitrogen species (RNS) and in the so-called nitrosative stress response [36]. The temporal coincidence of excess ROS and RNS production gives rise to the concomitant insurgence of oxidative/nitrosative stress [37]. In particular, the reaction between NO radical and superoxide anion (NO + O_2_•^−^) generates peroxynitrite (ONOO•^−^). Peroxynitrite decays into various unstable RNS, as well as into nitrite and nitrate, which are considered the stable NO end-products. Peroxynitrite and RNS actively take part in depleting cerebral antioxidant defences and in causing lipid peroxidation of mitochondrial membranes [38], further deteriorating Ca^2+^ homeostasis [39], and in the additional dysfunction of mitochondria through the formation of the membrane permeability transition pore [40], in delaying normalization of ionic homeostasis [41], and in mediating calpain-catalyzed proteolysis and neurodegeneration [42]. It is worth recalling that involvement of oxidative/nitrosative stress has been addressed as a fundamental pathobiological mechanism of cerebral tissue injuries occurring in numerous acute and chronic neurodegenerative disorders, affecting mitochondrial functions, and appearing particularly relevant in the progression of chronic neurodegenerative disorders (Alzheimer’s disease, Parkinson’s disease, multiple sclerosis) [43,44,45]. 

**Figure 5:**
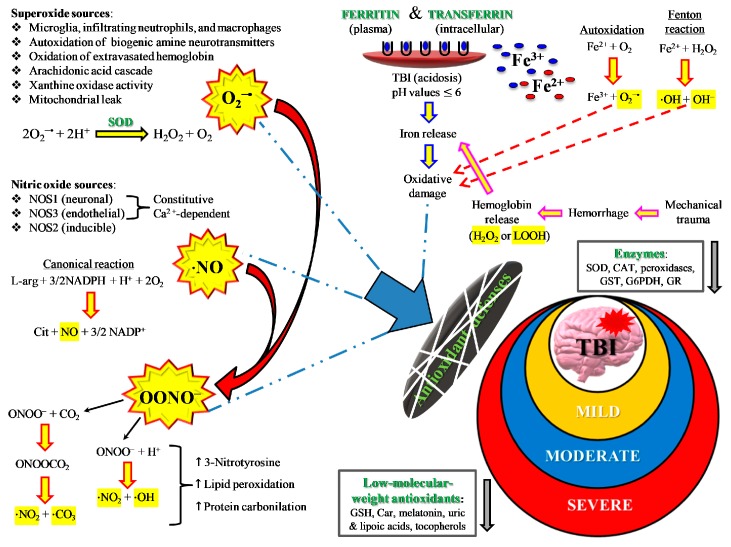
Schematic representation of the main sources of ROS and RNS during oxidative/nitrosative stress occurring after TBI. The severity of injury is quite well correlated with high or low oxidative/nitrosative stress and with protraction of intra- and extracellular ROS and RNS generation from the various potential sources. For instance, mild TBI rarely causes hematomas with hemoglobin extravasation and mobilization of ferritin iron, thus strongly decreasing the amount of iron used in the Haber–Weiss-sustained Fenton reaction. Conversely, severe TBI induces long-lasting conditions of metabolic derangement due to mitochondrial dysfunction. A vicious cycle is formed between the increased energy requirement, either to satisfy repairing processes or to counteract dangerous phenomena (glutamate excitotoxicity, ionic homeostatic disequilibrium), and the damaging molecules originating from mitochondrial ETC unable to manage the tetravalent reduction of molecular oxygen to water minimizing superoxide formation.

By definition, an insurgence of oxidative/nitrosative stress takes place anytime cells/tissues/organs undergo an imbalance between ROS and RNS formation and their respective antioxidant defenses. In addition to enzymes capable to scavenging ROS (SOD, catalase, glutathione peroxidase, heme oxygenase, thioredoxin), cells are protected from oxidative/nitrosative stress by low molecular weight antioxidants [46], particularly when considering protection from certain ROS (hydroxyl radicals) and RNS (peroxynitrite). A schematic representation of the main sources of ROS and RNS during oxidative/nitrosative stress occurring after TBI is shown in Figure 5.

**Figure 6:**
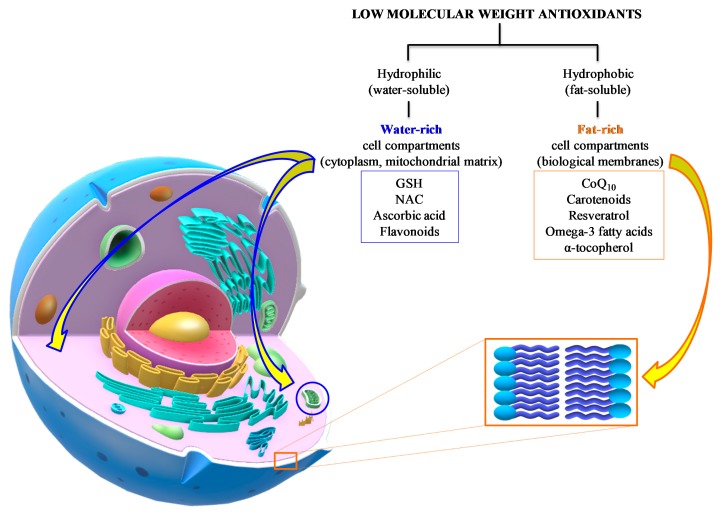
Summary of the main water- and fat-soluble antioxidants potentially useful as an adjuvant therapy to TBI patients. Their respective chemical properties localize the components of the two categories into hydrophilic (cytoplasm, mitochondrial matrix) or hydrophobic compartments (biological membranes), therefore characterizing the potential antioxidant activities of the different compounds.

In general, the common characteristic of these defense molecules is their remarkable reducing power that renders them highly “attractive” nearly for any type of oxidant, including ROS and RNS. A gross division into hydrophilic (water-soluble) and hydrophobic (fat-soluble) antioxidants clusters them into water-rich (cytoplasm, mitochondrial matrix) and fat-rich (biological membranes) cell compartments, thereby determining, and/or limiting, their possibility to interact with ROS and RNS (Figure 6). 

With the exclusion of reduced glutathione (GSH), uric acid, and coenzyme Q_10_ (these last two are secondary antioxidants since this is not their primary biological role), another common characteristic of low molecular weight antioxidants is that mammals are unable to perform their synthesis so that they depend on regular intake with diet to have adequate circulating and tissue concentrations of antioxidants [47]. Therefore, the quality of food consumed and/or supplementation of adjuvants and nutraceuticals is of fundamental importance in order to provide significant protection in the case of increased ROS and RNS formation [48,49]. Although the link between oxidative/nitrosative damage and TBI is evident and the awareness that deficiency in antioxidant-rich foods in the daily diet may further exacerbate TBI symptoms is highly plausible, there are still no clear evidence of successful antioxidant therapies in the clinical setting [50], despite a continuous growth of studies, reporting either preclinical or clinical data, using supplementation with natural or synthetic low molecular weight antioxidants, that have appeared in the literature in the last decades as potentially useful treatments in TBI [18,51]. This tendency is certainly due to different reasons, including the lack of standardized, clearly effective pharmacological approaches of TBI patients, the increased knowledge of the molecular mechanisms underlying cerebral damage after TBI, and the lack of unwanted side effects for the large majority of low molecular weight antioxidants.

In this review, we analyze data from the literature concerning the use of antioxidants in the post-injury period to alleviate the effects of TBI, considering either preclinical or clinical studies, with no limitations in terms of severity of TBI patients, experimental TBI models, animal species, routes of antioxidant administration, doses of antioxidant tested, parameters evaluated, but limiting our search only to those compounds having a well-established and direct antioxidant activity towards ROS and/or RNS.

## 3. Low Molecular Weight Antioxidants in TBI: Summary of Preclinical and Clinical Studies

### 3.1. Ascorbic Acid (Vitamin C)

Ascorbic acid (AA) is one of the most abundant water-soluble antioxidants within mammalian tissues, acting as a reducing cofactor in several enzymatic reactions [52]. Being a powerful reducing agent, it quickly reacts, through one or two electron reactions, with a wide range of ROS and RNS, including peroxynitrite and hydroxyl radicals. AA is involved in the recycling of oxidized -tocopherol (Vitamin E) contributing to the maintenance of -tocopherol redox state within biological membranes, and its deficiency is associated with impairments in connective tissue integrity. Since AA is not synthesized by most mammals (including human beings), the body depends on vitamin C-rich food ingestion to satisfy its AA need [53]. Efficient absorption of this molecule mainly occurs through the SVCT1 Na^+^-ascorbic acid transporter activity [54,55]. Although, circulating levels of AA are relatively low (30 to 70 mol/L serum in healthy humans) [47], abundant AA concentrations are found in peripheral tissues, particularly in the brain, where it is usually present in concentrations up to 3000 mol/L brain water [56,57,58]. The SVCT2 Na^+^-ascorbic acid transporter, ubiquitously distributed on the cell membrane of the different tissues, provides the electrogenic AA transport exploiting the favorable extracellular/intracellular sodium gradient [59,60]. Although AA is one of the most studied free radical scavengers and is particularly abundant in the brain, only one preclinical and one clinical study have examined the effects of AA in TBI, even though it has been shown that the level of AA in the cerebral tissue decreases rapidly following experimental TBI [56,61]. Its depletion is strictly dependent on the severity of the injury, remaining well below control values even longer after severe TBI and returning to pre-impact concentrations after 72 h in mildly injured animals [21,62]. In a model of closed-head TBI, animals received pre-treatment with different doses of AA (intraperitoneal administration of 45 or 60 mg/kg/die) for two weeks, alone or in combination with equal dosages of -tocopherol, that was administrated for an additional 2 weeks after impact [62,63]. Results of this study indicate that AA, even if administered alone, reduced mortality rate, decreased cerebral tissue and circulating levels of malondialdehyde (MDA), restored brain values of AA, and stimulated tissue superoxide dismutase levels [64]. 

In a double blind controlled clinical trial, Razmkon et al. [65] divided a cohort of 100 TBI patients into four groups receiving either a low dose of AA (500 mg/die i.v. for 7 days), or a high dose of AA (10 g i.v. on admission and 4 days after, followed by 4 g/die i.v. for the remaining 3 days), or vitamin E (400 IU/die i.m. for 7 days), or placebo. Beneficial effects of high dosage of this antioxidant were observed in AA-treated patients who showed decreased progression of perilesional edema on CT scan. The relatively modest effects of AA recorded in this study might be due to the malfunction of the SVCT2 transporter that normally ensures the electrogenic AA transport into cells. Potentially, the ionic imbalance typically encountered following TBI might perturb the functioning of the AA transporter, thus limiting the effectiveness of AA-based therapy. It might also be possible that decreased expression of SVCT2 occurs following TBI.

### 3.2. N-Acetyl-Cysteine

GSH is one of the low molecular weight antioxidants that mammals are able to synthesize [66]. Since the -SH group of GSH is supplied by cysteine, high levels of this amino acid are essential to ensure adequate GSH tissue levels. Due to the fundamental role of GSH in detoxifying reactions, both to scavenge ROS and RNS and to eliminate xenobiotics, increased cysteine availability is needed under conditions of oxidative/nitrosative stress. GSH functions as an essential antioxidant, as well as the most important reducing agent for protein –SH groups, and it has recently been demonstrated to be involved in the modulation of inflammatory processes [67]. It is the cofactor of the enzymes GSH peroxidases (GPx) and glutathione-S-transferases. The reduction of oxidized GSH (GSSG) is performed by the NADPH-dependent enzyme GSH reductase (GR), responsible for the maintenance of high values of the GSH/GSSG ratio. The brain is highly rich in GSH (3000 mol/L brain water), where it has been shown that GSH binds to the glutamate NMDA and AMPA receptors, possibly acting as an endogenous neuromodulators [68,69]. Additionally, GSH operates as an activating agent of ionotropic receptors, thus suggesting even a role of GSH in cerebral neurotransmission [70]. It is worth underlining that part of the total GSH cellular content is under the form of the stable S-nitrosoglutahione (GSNO) deriving from the interaction of NO with GSH. GSNO is considered a reservoir of intracellular NO, a transducer of the NO signaling, and an antioxidant due to its ability to break the lipid peroxidation reaction chain. GSNO levels are regulated by the NADH-dependent activity of GSNO-reductase, deeply involved in the regulation of protein nitrosylation and secondary formation of RNS [71]. Hence, maintaining high levels of GSH allows the formation of adequate concentrations of GSNO. Several experimental studies have observed a decrease in brain GSH following TBI. Among these, Ansari et al. showed a significant decrease of GSH/GSSG from 3 to 96 h post-TBI in rat hippocampus, which was mirrored by a time-dependent decline of GPx and GR activities [72]. It was reported that concentrations of rat brain GSH significantly declined depending on the severity of injury in a model of graded diffuse TBI [56]. Whilst in mild TBI (mTBI), a spontaneous recovery late post-injury was observed, severely injured animals had a permanent decrease of brain GSH up to a week following TBI [56]. In addition, a decrease of GSH in CSF of children who suffered a severe TBI has also been reported [73]. Similarly, lower levels of cysteine and glycine, both fundamental for the GSH biosynthesis, were also demonstrated after mild and severe TBI [74]. Interestingly, it has been demonstrated that the i.p. administration of 150 mg/kg γ-glutamylcysteine ethyl ester (GCEE), approximately 10 min postinjury, produced a significant decrease of oxidative/nitrosative damage to proteins following TBI in the rat [75], thus supporting the rationale of applying therapies aimed at increasing cerebral GSH of the post-injured brain.

The aforementioned results motivated the use of N-acetylcysteine (NAC) as a potential new treatment in TBI, either acting as a direct ROS and RNS scavenger, or operating by improving the availability of cysteine and ultimately enhancing GSH synthesis. Since these NAC effects have been well documented in both preclinical and clinical studies, various authors evaluated the benefits of NAC administration in experimental models of TBI, as well as its administration in TBI patients. The potential of GSH precursors as treatment options for TBI has been summarized in a recent review by Koza et al. [76]. In a model of contusion cortical impact, administration of a single dose of NAC (150 mg/kg) 15 min after injury decreased MDA formation, increased SOD and GPx, and protected morphology and the number of neurons of rats sacrificed at 2 and 12 h post-TBI [77]. NAC was also used in combination with minocycline (MINO) and administered 12 h after injury. NAC + MINO prevented the TBI-induced decrease in the expression of oligodendrocyte antigenic markers CC1, 2′,3′-cyclic-nucleotide 3′-phosphodiesterase, and phospholipid protein between 2 and 14 days post-CHI [78]. These results indicated an ample therapeutic window of NAC administration justifying the potential clinical application of NAC in the treatment of TBI patients. In a different study, 150 mg/kg NAC was orally administered (via gastric gavage) at 1, 24, 48, and 72 h after mild closed-head TBI. Results showed that NAC decreased plasma lipid peroxidation and IL-1b levels, increased brain cortex IL-4, GSH, TAS, vitamin A, AA and -tocopherol values, and increased erythrocyte GSHPx levels [79]. 

### 3.3. Flavonoids

Flavonoids are a class of more than 5000 naturally occurring compounds, largely present in plants and fungi [80]. Their common structure is formed by one phenyl ring fused with an oxygen-containing heterocyclic ring to which is linked a second phenyl ring. Since, in the different flavonoids, the number of –OH groups is higher than one, flavonoids are more generally termed as polyhydroxy polyphenols, or simply polyphenols. Chemically, they are divided into three groups: flavonoids or bioflavonoids, isoflavonoids, and neoflavonoids. Flavonoids are divided into five sub-groups (anthocyanidins, anthoxanthins, flavanones, flavanonols, and flavans), isoflavonoids into five sub-groups (isoflavones, isoflavonones, isoflavans, pterocarpans, rotenoid), and neoflavonoids into two sub-groups (neoflavones and neoflavenes). The large majority of all the aforementioned compounds are water-soluble, and, it is assumed, they are consumed in variable proportions, with foods of vegetal origin, according to the different dietary habits. The common characteristic of flavonoids is that they possess a very negative oxido-reductive potential, therefore acting as powerful antioxidants towards any type of ROS and RNS [81]. A growing number of preclinical studies have investigated the effects of flavonoid administration in reducing TBI-associated damage, the results of which have been condensed into Table A1 of Appendix A. 

Given the wide interest in the assessment of natural compounds (including flavonoids) obtained from a variety of vegetable sources and the large number of flavonoids, it is difficult to find two studies examining the same flavonoid, and it is therefore difficult to directly compare studies investigating the effectiveness of flavonoid-based therapies.

In a small pilot clinical trial, 60 patients who experienced mild TBI and showing symptoms from 3 to 12 months after injury, received oral enzogenol (1 g/die) for 6 weeks. Cognitive functioning before and after treatment was investigated. Compliance, side-effects, cognitive failures, working and episodic memory, post-concussive symptoms, and mood were assessed at baseline, 6, 12, and 16 weeks. Patients receiving enzogenol were found to have significantly fewer self-reported cognitive failures than those in the placebo group [82]. 

### 3.4. Resveratrol

Resveratrol is an antioxidant with modest, but significant, solubility in water and good solubility in organic solvents, thus indicating its prevalent hydrophobic nature. It is formed by two 6-carbon aromatic rings (one with two and the other with one -OH groups) linked by a C=C bridge and allowing the existence of cis- and trans-resveratrol. This form of polyphenol is abundantly present in the skin of red grapes. However, red wines are the food source with the highest resveratrol levels. High resveratrol intake, due to high red wine ingestion, was originally suggested as one of the possible explanation of the so-called “French paradox” concerning the apparently low occurrence of cardiovascular diseases in the French population, in spite of a high fat (mostly saturated) diet consumption [83]. Apart from its intrinsic antioxidant activity, resveratrol seems to modulate several cell functions, including defense mechanisms, mitochondrial functions, and inflammatory processes [84].

A preclinical study in rats evaluated the effects of a single dose of resveratrol (100 mg/kg) administered intraperitoneally immediately after TBI induced by the weight-drop model. Brain water content and levels of MDA, GSH, NO, and xanthine oxidase (XO) were evaluated at 24 h after impact. In the resveratrol-treated group, significantly lower values of MDA, XO and NO, and increased level of GSH were observed. Additionally, resveratrol treatment also attenuated tissue lesion area [85]. Another study analyzed the protective effect of resveratrol in TBI in vivo and in vitro. Results showed that cell death after TBI is induced through the ROS/GSK-3β/mitochondria signaling pathway and that administration of resveratrol (100 mg/kg) immediately after controlled cortical impact (CCI)–TBI can increase cell survival by suppressing GSK-3β-mediated autophagy and apoptosis [86]. In one additional study, mice were treated with either placebo or resveratrol (100 mg/kg) at 5 min and 12 h after induction of mild TBI using CCI. In comparison to placebo-receiving animals, results demonstrated a reduction of microglial activation in the brain regions of the cortex, corpus callosum, and dentate gyrus induced by mild TBI in resveratrol treated rats. In addition, resveratrol decreased IL-6 and IL-12 expressions suggesting a general decrease of neuroinflammation caused by TBI [87].

### 3.5. α-Tocopherol (Vitamin E)

Tocopherols are a family of fat-soluble compounds, found in high concentrations in various vegetal oils, having a remarkable antioxidant capacity. Tocopherol congeners having vitamin E activity are four tocopherols and four tocotrienols, the former having a saturated hydrocarbon harbored side chain, the latter having an unsaturated hydrocarbon harbored side chain composed by three isoprene units [88,89]. The chromane ring, to which the hydrocarbon chain is bound, is the oxido-reductive center common to all the eight compounds. Its single OH-group can easily lose one electron, in the form of a hydrogen radical, under the action of highly oxidizing ROS and RNS and forming the corresponding, relatively stable, tocopheryl- or tocotrienyl-radical. The hydrophobic side chain of tocopherols allows these compounds to localize within the phospholipid bilayer of biological membranes, where they act as interrupters of lipid peroxidation chain reactions. When oxidized by ROS and RNS, tocopheryl-radicals strictly depend on AA availability to get recycled into their respective fully reduced forms. Among the eight congeners, -tocopherol is the most abundant in the European diet and -tocopherol in the American diet. Albeit the brain is rich in easily oxidizable fat-soluble compounds, mainly found in the biological membrane structures of the nerve cells, it is one of the mammalian tissues with the lowest content of tocopherols. This renders the brain potentially more exposed to lipid peroxidation phenomena mediated by oxidative/nitrosative stress.

As in the case of AA, several animal studies using different TBI models at different severities have been conducted, but only one clinical study evaluated the effects of -tocopherol administration to TBI patients. In guinea pigs experiencing mild or severe TBI, Inci et al. found that -tocopherol (100 mg/kg), intraperitoneally administered before graded TBI, decreased brain levels of MDA derived from oxidative/nitrosative stress-mediated lipid peroxidation. The authors concluded that -tocopherol administered early post-injury is beneficial to decrease tissue damage associated with TBI [90]. In a model of focal moderate TBI, Sprague–Dawley rats received a daily intraperitoneal injection of -tocopherol (600 mg/kg), subsequently to TBI induction. At different times post-impact, neurocognitive tasks, histochemical evaluation of tissue damage, and expressions of Nogo-A and NgR, two protein factors inhibiting nervous cell regeneration, were evaluated in all animals. Results showed that rats treated with -tocopherol performed better on neurocognitive tests, had less evident histological signs of edema, inflammation, and necrosis, as well as decreased expression of Nogo-A and NgR. The authors proposed that -tocopherol may reduce ROS-mediated tissue damage and promote cerebral tissue regeneration following TBI [91]. In a model of focal moderate TBI, the effects of administration of tocopheryl-succinate (100 mg/kg) + polyethylene glycol (PEG) (2 mL/kg), injected 30 min before or 5 min after injury, were evaluated in rats subjected to fluid percussion. Although PEG alone improved the parameters under evaluation, the combination tocopheryl-succinate (100 mg/kg) + PEG (2mL/kg) showed the best increase in the survival rates and the best improvement of neurocognitive tasks and motor function, suggesting that this type of infusion might be effective in the clinical setting to decrease TBI-associated damage [92]. Aiguo et al. fed rats using a regular diet with or without 500 IU/kg of -tocopherol for 4 weeks, at the end of which animals received focal mild TBI, according to the fluid percussion injury model [93]. It was shown that the -tocopherol-rich diet prevented TBI-increased oxidative damage to proteins, the decrease in SOD, Sir2, and BDNF caused by TBI, and improved TBI-associated motor function impairments. The authors’ conclusions were that dietary -tocopherol supplementation could decrease the damaging effects of mild TBI on synaptic plasticity and cognitive functions.

Despite the beneficial effects of -tocopherol in preclinical studies, translation to the clinical setting was only performed in the previously cited Razmkon study [65]. Among the four groups of TBI patients, those who were administered -tocopherol (400 IU/die intramuscularly for 7 days) showed a significant reduction of mortality rates and improvement of long-term functional outcomes. 

### 3.6. Coenzyme Q_10_

Coenzyme Q (ubiquinone) is a fat-soluble compound ubiquitously found as a component of the mitochondrial electron transport chain in oxygen-dependent living organisms. It is characterized by a quinone ring which, through oxido-reductive reactions exchanging one or two electrons, allows coenzyme Q to exist in a fully oxidized, fully reduced, or semi-reduced (semi-quinone radical) form. Depending on the organism considered, the quinone ring is bound to a hydrophobic chain, made up of isoprene units, repeated from a minimum of 3 to a maximum of 10 units (as it occurs in humans). This last form of coenzyme Q, named coenzyme Q_10_ (CoQ_10_), is found in the mitochondrial inner membrane actively participating in the transfer of electrons from Complex I and Complex II to Complex III, where the so-called CoQ_10_ cycle takes place [94]. 

Several studies were performed, testing the effects of exogenously administered CoQ_10_ under various pathological conditions to counteract oxidative/nitrosative stress, ameliorate mitochondrial dysfunction, and decrease inflammatory processes [95]. The rationale was attributed to the CoQ_10_ oxido-reductive potential, by its ability to affect one-electron transfer reactions by forming the semi-quinone radical, by the relatively long-living, low reactivity of its semi-quinone radical and by its high hydrophobicity clustering CoQ_10_ within biological membranes. It should, however, be recalled that the main CoQ_10_ biological role is that of ensuring the electron transfer through ETC and not that of acting as an antioxidant [96]. However, very recently, it has been shown that CoQ_10_ interacts with FSP1 acting as a novel plasma membrane oxidoreductase and protecting cells from glutathione-independent ferroptosis [97].

At present, few animal studies have evaluated the role of CoQ_10_ administration in the treatment of head trauma, with no clinical studies conducted to date. In the Marmarou model of moderate diffused closed-head TBI, CoQ_10_ was administered at a dose of 10 mg/kg immediately after trauma and after 24 h by gavage. Compared to trauma only, this treatment decreased MDA levels, vascular congestion, neuronal loss, nuclear pyknosis, nuclear hyperchromasia, cytoplasmic eosinophilia and axonal edema, and increased cerebral SOD [98]. In rats receiving TBI according to CCI, administration of CoQ_10_ immediately after impact was found to significantly affect the cerebral expression of genes involved in mechanisms of bioenergetics and oxidative/nitrosative stress [99]. The same research group tested the effects of intra-arterial CoQ_10_ infusion before or 30 min after TBI induced by CCI [100]. Results indicated a decrease in brain mitochondrial damage and apoptosis, as well as lower circulating values of two markers of TBI severity, namely, serum glial fibrillary acidic protein (GFAP) and ubiquitin C-terminal hydrolase-L1 (UCH-L1). However, in this study, in vivo MRS (magnetic resonance spectroscopy) failed to show any significant amelioration of neurometabolism produced by CoQ_10_ treatment [100].

### 3.7. Carotenoids

Carotenoids are a class of pigments synthesized by plants, algae, and photosynthetic bacteria [101], composed of 8 isoprene units with a total of 40 carbon atoms. There are over 1100 known carotenoids, which are categorized into a class of carotenoids containing oxygen (xanthophylls) and a class of oxygen-free, purely hydrocarbon carotenoids (carotenes). In the human brain, various carotenoids, including -carotene, -carotene, -carotene, -cryptoxanthin, lutein, and zeaxanthin, can generally be measured [102]. Some of them (-carotene, -carotene, -carotene, -cryptoxanthin) can be converted into vitamin A and are therefore directly involved in the mechanism of vision, ensuring adequate retinol formation. Since the different carotenoids are distributed into a variety of foods mainly of vegetal origin (fishes and crustaceans, such as salmon and red shrimps, derive their high carotenoid level on the high intake of carotenoid-containing algae), their daily ingestion, and therefore their relative amount within tissues, is strictly dependent on the individual dietary regimen, as well as on the supplementation of carotenoid-containing adjuvants or nutraceuticals. In general, carotenoids exhibit antioxidants and anti-inflammatory properties, as well as possess modulatory activities of autophagy. The use of various carotenoids as potential treatments for several pathologies, including neurodegenerative diseases, have been extensively studied [102]. 

In a stretch injury model of TBI using astrocyte cultures, astaxanthin attenuated apoptosis by inhibiting NKCC1 expression, and reduced the expression of NF-κB-mediated pro-inflammatory factors [103]. In mice, intraperitoneal injection of astaxanthin (10, 25, 50, or 100 mg/kg body weight) administrated 30 min after impact, decreased TBI-related brain tissue injury (induced by CCI) by dose-dependently ameliorating AQP4/NKCC1-mediated cerebral edema. In addition, the highest dose examined was shown to improve neurologic deficits and the BBB permeability [104]. In a different study, astaxanthin (25 or 75 mg/kg) was administered to mice via oral gavage beginning 30 min post-trauma and followed by six additional daily oral gavages. Animals received a left frontal TBI using a weight-drop device. Results showed that astaxanthin administration improved sensorimotor performance and enhanced cognitive recovery. In addition, reduction of lesion size in the cortex and expressions comparable to controls of brain-derived neurotrophic factor (BDNF), growth-associated protein-43 (GAP-43), synapsin, and synaptophysin (SYP), indicating the induction of neuronal survival and plasticity, were recorded [105]. 

Many other carotenoids have been investigated in TBI animal models, and molecular mechanisms have also been described. Among these, fucoxanthin showed neuroprotective effects via regulation of the Nrf2-ARE and Nrf2-autophagy pathways [106]. Bexarotene inhibited apoptosis and inflammation by upregulating the lncRNA Neat1 [107], and improved the neurological functions of mice after TBI, partially through apolipoprotein E [108]. Administration of crocin has shown neuroprotective effects partially dependent on the activation of Notch signalling [109]. Lastly, lutein, another strong antioxidant with anti-inflammatory and anti-apoptotic effects, and capable of crossing the BBB, showed protective effects by suppressing interleukin IL 1β, IL 6, and monocyte chemoattractant protein 1 expressions [110].

### 3.8. Omega-3 Fatty Acids

Omega-3 fatty acids are a group of polyunsaturated fatty acids (PUFA) that have three (linolenic acid) to six (docosohexaenoic acid) double bonds in their carbon chain of 18 (linolenic acid) to 22 (docosohexaenoic acid) carbon atoms in length. Considering their last carbon atom as the -atom, this group of PUFA has the common characteristic of one double bond positioned on the third carbon atom far from the -atom. Omega-3 fatty acids are either essential (linolenic acid) or semi-essential (eicosapentaenoic acid, docosahexaenoic acid) for humans. Our body is unable to synthesize linolenic acid (the precursor of the other omega-3 fatty acids) that should be ingested in adequate amounts with diet. However, humans are capable of providing little but significant amounts of docosahexaenoic acid (DHA) and eicosapentaenoic acid (EPA) from the elongation and desaturation of linolenic acid, through a specific metabolic pathway. Omega-3 fatty acids are not only fundamental components of membrane phospholipids but also precursors of other important biologically active molecules (prostaglandins). Particularly DHA and EPA are abundantly found in the brain phospholipids, with the former representing about 50% of the weight of the neural tissue cell membranes [111]. Although real benefits in patients with neurodegenerative disorders are still controversial, the supplementation of omega-3 fatty acids has been tested in various pathologies characterized by increased ROS and RNS production. The rationale is either in the pure antioxidant activity of omega-3 fatty acids or in the possibility that an aliquot of those exogenously administered might help in restoring biological membranes damaged by oxidative/nitrosative stress-mediated lipid peroxidation.

In Table A2 of Appendix A, we summarize key preclinical studies evaluating the role of omega-3 fatty acids and DHA in TBI and examined more detailed clinical studies carried out in TBI patients. It is worth underlining that the three clinical studies available in the literature do not support the results reported in experimental TBI. 

Matsouoka et al. [112] evaluated the effects of the combined daily administration for 12 weeks of 1470 mg DHA and 147 mg EPA to a group of 53 traumatized patients, 52% of which suffered from mild TBI. A control group of 20/57 traumatized subjects sustained mild TBI and was administered with placebos. Outcome measures were posttraumatic stress disorder (PTSD) symptoms, assessed by the Clinician-Administered PTSD Scale, and depressive symptoms, assessed by the Montgomery–Åsberg Depression Rating Scale, both evaluated at baseline and after 12-weeks, together with the serum levels of mature BDNF and precursor pro-BDNF. Results showed no effect of DHA + EPA on PTSD, depressive symptoms, or serum BDNF levels, thus confuting the efficacy of these omega-3 in ameliorating mental status of patients sustaining mild TBI [109]. Same authors reported no effects of the combined daily administration for 12 weeks of 1470 mg DHA and 147 mg EPA, even if enriched with 0.3% a-tocopherol, when the same patients were fully evaluated for the post-injury quality of life. Also in this case, the authors concluded that the administration of omega-3, specifically DHA and EPA, is not justified by their clinical results to treat TBI patients [113]. The last study showed a clinical case of an elderly patient taking warfarin and fish oil supplementation (rich in omega-3 fatty acids) after suffering blunt head trauma and who manifested an irreversible fatal warfarin-induced coagulopathy. This study, although reporting only one case, reveals the risks of omega-3 fatty acid supplementation in patients taking anticoagulant medications, because of the synergistic effect of the two treatments (warfarin and omega-3 fatty acids) in inhibiting platelet aggregation promoting untreatable bleeding [114]. 

## 4. Discussion

Concussion is a traumatically induced transient perturbation of brain functions occurring at any time impacts of different intensities, not necessarily hitting the head, cause an acceleration–deceleration phenomena of the skull [115,116]. The most dangerous impacts are those inducing rotational and translational movements of the head. Very commonly, and erroneously, the term concussion is interchangeably used with mild TBI. Therefore, it is certainly true that all concussions are mTBIs, and it is equally true that not all mTBIs are concussions [117]. Concussions are characterized by a rapid onset of spontaneously resolving clinical symptoms, typically represented by neurological impairment and cognitive deficits. Since during a concussive event part of the energy associated to the impact is also discharged towards the cerebral tissue, concussion is known to trigger a plethora of biochemical, metabolic, and molecular changes transiently, but deeply, altering cerebral cell functions and homeostasis. This complex pathobiological process is also known as the neurometabolic cascade of concussion [117,118,119]. The concussed brain is characterized by transient alteration of ionic homeostasis, imbalanced cellular calcium exchange and storage, which leads to mitochondrial dysfunction with an energy crisis. Glucose dysmetabolism [120], alteration of the mitochondrial quality control [121,122], impairment of cell energy state [121,122,123], and increase of ROS and RNS production [124] characterize a temporal window following concussion during which the brain is “metabolically vulnerable” It has been clearly demonstrated that alterations of brain metabolism last much longer than symptom disappearance and return of neurocognitive functions to pre-impact levels. A second concussive event of even lesser magnitude than the first, falling within this period of vulnerability, may have catastrophic consequences causing a clinical condition termed as second impact syndrome (SIS). When occurring, SIS is fatally characterized by untreatable malignant cerebral edema associated with high disability and mortality rates. Repeat concussions, taking place before complete restoration of neurometabolism, may be the basis of the degeneration into chronic traumatic encephalopathy (CTE). CTE has been described in retired athletes who sustained a high number of concussions (mainly unreported) in their career [125], and it is a progressive neurodegenerative disorder causing a dramatic loss of neurocognitive functions and evolving into dementia [125,126].

Concussions represent about 20% of all mTBI. Of all concussions, 80% are sports-related concussions. Athletes, particularly those practicing sports characterized by frequent physical contact (such as boxing, martial arts, American football, ice hockey), are at much higher risks of concussions than non-athletes. Amateur and non-athletes suffer the influence of post-concussive symptoms in their everyday life as the main consequences connected to concussions. In contrast, the main problems for semi-professional and professional athletes refer to the time they are forced to stay out of the competitions, before their return to play following a concussion. According to what is stated above, sports-related concussions are a type of TBI in which prevention might effectively be applied either by modifying rules of those sports disciplines at higher risk of concussion, or in preventively treating athletes with drugs capable of inhibiting specific molecular pathways activated by concussions. It should also be taken into account that drug treatments might be helpful in allowing safer return of athletes to play. In this light, few studies have been carried out to evaluate the effects of the administration of antioxidants prior to concussion in reducing molecular changes and symptoms associated with concussion [127].

### Antioxidant Therapies in Sports-Related Concussion

In a clinical study evaluating the effect of enzogenol administration, 42 athletes with a history of sports-related concussion in the chronic phase of injury were enrolled. Athletes sustained concussions from 0.5 to 3 years prior to the start of the study and 28/42 of them experienced repeat concussions. They were blindly and randomly divided into two groups, one receiving placebos and the other 1 g/die of enzogenol. Both treatments were administrated for six weeks. Athletes were assessed on recruitment and at the end of drug administration, using virtual reality (VR), electroencephalography (EEG), and neuropsychological (NP) tasks. The enzogenol-treated group showed enhanced frontal-midline theta and decreased parietal theta power, indicating reduced mental fatigue compared to the placebo group. Subjects enrolled in the enzogenol group also self-reported reduced mental fatigue and sleep problems. The authors concluded that enzogenol has the potential to improve brain functioning in the chronic phase of concussion [128].

In a study evaluating the prophylactic effects of DHA administration, 81 American football athletes were recruited, and blindly and randomly administered with 2, 4, and 6 g/die of DHA or placebo. During the 189 days of the study duration, blood of athletes was analyzed at various times (coincident with changes in intensity, hours of contact, and likely changes in head impacts) neurofilament light (NFL) serum levels, as an indicator of axonal injury. The main finding was that DHA likely attenuates the increase in serum NFL, suggesting a neuroprotective effect of DHA towards a central pathogenic mechanism of concussion (axonal injury). Interestingly, the lowest dose (2 g/die) appeared to produce the most marked decrease in serum NFL levels [127].

Although it did not refer to sports-related concussions, one clinical trial (NCT00822263), carried out to test the efficacy of NAC on symptoms associated with blast exposure mild TBI (causing the same symptoms as concussion) in a Southwest Asia combat setting, is certainly worth being mentioned. In this prospective study, 81 symptomatic U.S. service members who were exposed to a blast wave or who were in a vehicle that was damaged by a blast wave, and who met the criteria for concussive mild TBI (balance dysfunction, confusion, headache, sensorineural hearing loss, impaired memory, and sleep disturbances), accepted to participate in the study. Randomly assigned patients received either placebo or 4-g loading dose of NAC followed by 4 g daily (in two divided doses of 2 gm morning and night) for 4 days and then by 3 gm daily (in two divided doses of 1.5 gm morning and night). Treatments started in a variable time ranging 24–72 h from blast exposure. When compared to placebo, results showed beneficial effects of oral NAC administration on neuropsychological test results, number of TBI symptoms, and full symptom resolution on day seven, following the beginning of treatment. Early initiation of NAC administration was also found to have additional benefit on the neurological but not on neuropsychological outcome measures [126].

## 5. Conclusions

The analysis of the literature concerning the beneficial effects of antioxidant-based therapies to reduce TBI-associated damage leads to the following indications: (i) there are no sufficient clinical data to determine whether adding the administration of antioxidants to that of drugs commonly used in TBI patients improves outcome in terms of decreased mortality and disability; (ii) for the majority of both water- and fat-soluble antioxidants, clinical studies are completely lacking; (iii) data from experimental TBI in laboratory animals are supportive of beneficial effects of administration of antioxidants in TBI of any severity; (iv) clear effectiveness of antioxidants, dosages, timing and route of administration, biochemical and molecular parameters, clinical and neurological parameters have not yet been established, rendering less strong the indications reported in these preclinical studies; (v) there are no data in the literature, either clinical or preclinical, for natural antioxidants (lycopene, anthocyanins) commonly found in a balanced diet, such as the Mediterranean diet. 

It is our opinion that it will be very difficult to obtain conclusive evidence showing that antioxidant administration to TBI patients is truly beneficial. In the past, preclinical studies demonstrated that ROS generation [129] and ROS-mediated damage [130,131] take place in the very early phase following TBI. Furthermore, a clinical study, in which TBI patients were analyzed for CSF content of antioxidants and ROS-mediated lipid peroxidation biomarkers within 3 h from injury, clearly showed that all TBI patients reached the NICUs, having dramatic ascorbate decrease and evident MDA increase in their CSF samples [132], i.e., most of ROS- and RNS-mediated damage was already completed at the time of potential antioxidant administration. Altogether, the aforementioned preclinical and clinical findings indicate a very narrow therapeutic window to allow antioxidant therapies to have a reasonable probability of having positive effects. It is also our opinion that it should be much more effective to test, either in animals or in human beings, the administration of a cocktail of antioxidants, distributing in different cell compartments, having differential ROS and RNS scavenging activity and performing secondary activations of different pathways beneficial for the post-injured brain, rather than expecting resounding ameliorations using the administration of one antioxidant only.

One further crucial observation is that, in the case of TBI, it is not feasible to attempt preventive medicine based on the administration of antioxidants, since there is no chance to know whether one will sustain TBI during his life span. Differently, preventive administration of antioxidants might be performed in sports-related concussion. By initially selecting those athletes practicing sports at high risk of concussion (any contact and some non-contact sports), it might certainly be easy to demonstrate whether athletes treated with antioxidants and who experienced a concussion have less concussion-associated symptoms, cognitive deficits, and neurometabolic alterations, as well as having shorter recovery periods than control, placebo-treated, concussed athletes. Therefore, it appears quite evident that sports-related concussion and athletes have the best chance of representing the preferential TBI type and patient population to evaluate future antioxidant therapies in humans. 

## Figures and Tables

**Table 1:** Traumatic brain injury (TBI) severity classification, according to the Glasgow Coma Scale (GCS). The score for each patient is calculated by summing the points obtained in each set of neurological examination. Therefore, the minimal value that a TBI patient may score is 3 (corresponding to comatose severe TBI patients) and the maximal is 15 (corresponding to the mildest group of mild TBI patients).

Score	Eye Opening (E)	Verbal Response (V)	Motor Response(M)
1	No eye-opening	No verbal response	No response
2	Eye-opening to pain	Incoherent	Extension to pain
3	Eye-opening to speech	Inappropriate words	Flexion to pain
4	Spontaneous eye-opening	Confused conversation	Withdrawal to pain
5		Oriented	Localizes to pain
6			Follows commands

**Table 2:** Summary of the main drug treatments administered to stabilize clinical conditions of TBI patients in emergency departments.

Class	Treatment Drugs	Mechanism of Action
Osmotic therapy	Mannitol Hypertonic saline	Decrease brain edema, improve cerebral blood flow and blood rheology
Antiepileptic drugs	Phenytoin, Phenobarbital, Carbamazepine, Valproate, Levetiracetam	Prevent seizures, especially during the first week after injury thus preventing rise in ICP
Sedative agents	Barbiturate: Pentobarbital Benzodiazepine: Midazolam	Reduce neuronal activity, metabolic brain requirements and ICP
Pharmacological paralysis	Succinylcholine, Atracurium, Rocuronium	Prevents high intra-thoracic pressure during mechanical ventilation which is transmitted intra-cranially
Opioid analgesics	Morphine, Fentanyl, Alfentanil	Pain control via their action on neuronal opioid receptors

## Abbreviations

AA	Ascorbic Acid
AMPA	α-amino-3-hydroxy-5-methyl-4-isoxazolepropionic acid
AQP4	Aquaporin-4
BBB	Blood–Brain Barrier
BDNF	Brain-Derived Neurotrophic Factor
CBF	Cerebral Blood Flow
CBV	Cerebral Blood Volume
CCI	Controlled Cortical Impact
COX	Cyclooxygenase
CSF	Cerebro-Spinal Fluid
CT	Computed Tomography
CTE	Chronic Traumatic Encephalopathy
DHA	Docosohexaenoic Acid
DHE	Dihydroethidium Staining
DRP1	Dynamin-Related Protein 1
EEG	Electroencephalography
eNOS	Endothelial Nitric Oxide Synthase
EPA	Eicosapentaenoic Acid
ETC	Electron Transfer Chain
FIS1	Fission Protein 1, mitochondrial
FJB/C	Fluoro Jade B or C staining
FST	Forced Swimming Test
FPI	Fluid Percussion Injury
GAP-43	Growth-Associated Protein-43
GCS	Glasgow Coma Scale
GFAP	Glial Fibrillary Acidic Protein
GPx	Glutathione Peroxidases
GSH	Reduced Glutathione
GSK-3β	Glycogen Synthase Kinase 3 Beta
GSNA	Greater Splanchnic Nerve Activity
GSNO	Nitrosoglutathione
GR	GSH Reductase
GSSG	Oxidized Glutathione
Hb	Haemoglobin
H&E	Hematoxylin and Eosin staining rotocol
ICH	Immunohistochemistry
ICP	Intracranial Pressure
IL	Interleukin
iNOS	Inducible Nitric Oxide Synthase
i.m.	Intramuscular
i.v.	Intravenous
lncRNA Neat1	Long noncoding RNA nuclear enriched abundant transcript 1
MDA	Malondialdehyde
MFN1 or 2	Mitofusin 1 or 2
MINO	Minocycline
mNSS	Modified Neurological Severity Score
mTBI	Mild Traumatic Brain Injury
MWM	Morris Water Maze
NAC	N-Acetylcystein
NFL	Neurofilament Light
NF-κB	Nuclear Factor kappa-light-chain-enhancer of activated B cells
NICUs	Neurosurgical Intensive Care Units
NKCC	Sodium–potassium–chloride cotransporter
NMDA	N-Methyl-D-Aspartate
nNOS	Neuronal Nitric Oxide Synthase
NO	Nitric oxide
NOR	Novel Object Recognition
NOS	Nitric Oxide Synthases
NOX	NADPH Oxidase
NP	Neuropsychological
Nrf2-ARE	Nuclear factor E2-related factor 2 Antioxidant Response Elements
NSS	Neurological Soft Signs
OPA1	Optic dominant Atrophy 1
OXPHOS	Oxidative Phosphorylation
PARK2	Parkin
PAT	Passive Avoidance Task
PEG	Polyethylene Glycol
PINK1	mitochondrial serine/threonine-protein kinase 1
PTSD	Posttraumatic Stress Disorder
PUFA	Polyunsaturated Fatty Acids
RNS	Reactive Nitrogen Species
ROS	Reactive Oxygen Species
SIS	Second Impact Syndrome
SOD	Superoxide Dismutase
SVCT1 or 2	Sodium dependent vitamin C transporter 1 or 2
SYP	Synaptophysin
TAC	Total Antioxidant Capacity
TBARS	Thiobarbituric Acid Reactive Substance
TBI	Traumatic Brain Injury
TEM	Transmission Electron Microscopy
TST	Tail Suspension Test
TUNEL	Terminal deoxynucleotidyl transferase (TdT)-mediated dUTP nick-end labeling assay
UCH-L1	Ubiquitin Carboxy-terminal Hydrolase-L1
VCS	Veterinary Coma Scale
VR	Virtual Reality
WB	Western Blot
WD	Weight Drop
XO	Xanthine Oxidase

## Appendix A

**Table A1:** Summary of preclinical studies on flavonoid administration in different models of TBI.

Name of Flavonoid	Dosage and Route of Administration	Time Points	Tests and Assays	TBI Model	Main Findings	Ref
Chrysin	25, 50, or 100 mg/kg orally, started immediately post-injury and continued for up to 3 or 14 days	1 day before injury; 0, 1, 4 h, and 1, 2, 3, 4, 7, 13, 14, 28 days post-injury	VCS, rotarod test, PAT, biochemical assay, histochemical staining, TUNEL, IHC	WD	Chrysin enhanced post-injury motor function, cognitive status and neuronal loss via reduction of oxidative stress (increased concentrations of SOD, CAT, GPx, GSH, and decreased MDA level) and inhibition of the apoptotic proteins (decreased Bax and increased Bcl-2) in the cerebral cortex and CA3 hippocampal neurons	[133]
Wogonin	1, 2.5, 5 mg/kg. (i.v.) before and after moderate TBI; Alternatively, 2 µL (10 mM) injected into the lateral cerebral ventricle (i.c.v.) before and after moderate TBI	Before injury and 0.5, 1, 2, 4 h post-injury	Arterial pressure, heart rate, baroreflex and GSNA, histochemical staining of hippocampus	FPI	Wogonin administered before or after TBI significantly improved the cardiovascular changes (MAP, HR, and baroreflex) occurring after FPI.	[134]
Isoliquiritigenin (ILG)	20 mg/kg (i.p.) 1 h post-TBI	1 and 7 days after TBI	Sensorimotor Garcia test, brain water content, BBB permeability assay, histochemical staining, cell viability assay, WB, RT–qPCR, fluorescence immunoassays	CCI	ILG improved neurologic functions by reducing brain edema, BBB permeability, apoptosis (decrease in expression of cleaved caspase 3), and oxidative stress (translocation of Nrf2 into the nucleus with activation of downstream proteins).	[135]
Hesperidin	50 mg/kg orally from day 10 to 24 post-injury	Day 21 and 24 post-injury	Sucrose preference test, FST, suppressed feeding test, TST, biochemical analysis	WD	Hesperidin reduced depression symptoms, levels of IL-1β, TNF-α and MDA, and increased BDNF levels in the hippocampus	[136]
Icariin	Oral administration of 3, 10, 30 mg/kg on injury induction and daily for 7 days post-injury	0, 3, 7, 8 days post-TBI	Rotarod test, balance beam test, Y-maze, WB, IHC, histochemical staining, protein expression	Modified CCI	Icariin improved sensory motor and cognitive functions, and upregulated BDNF, SYP, and PSD-95 after injury. However, no improvement of brain histology and neuronal death were found.	[137]
Baicalin	50, 100, 150 mg/kg (i.p.) 30 min after TBI	2 h, and 1 and 3 days post-TBI	NSS, brain water content, TUNEL assay, WB Immunostaining, RT-qPCR	WD	Baicalin improved neurological function, brain edema, apoptosis, and oxidative stress via activating the Akt/Nrf2 pathways	[138]
Formononetin	Intragastrical administration of 10, 30 mg/kg/die for up to 7 days after TBI	7 days post-injury	ELISA of tissue and serum cytokines, Cytohistologic stains, RT-qPCR, WB	WD	Formononetin counteracted TBI-induced neuroinflammation by decreasing tissue and serum levels of IL-6 and BDNF, and increasing tissue and serum levels IL-10	[139]
Troxerutin	1.5 mL/kg (i.p.) for 5 days before TBI	3 days post-injury	NSS, MRI, ELISA, Nissl, TEM, WB and Immunostaining	WD	Troxerutin improved neurovascular function and integrity post-injury through action on eNOS and NO level, with consequent decrease in peroxynitrite formation	[140]
Quercetin	50 mg/kg (i.p.) at 30 min, 12 h and 24 h post-TBI	1, 3, 5 days post-injury	Brain water content, NSS, H&E staining and neuron count, IHC, WB	WD	Reduced brain edema, neural apoptosis and improved motor function (inhibition of extracellular signal-regulated kinase 1/2 phosphorylation and activated Akt serine/threonine protein kinase phosphorylation)	[141]
Resveratrol	0.05, 0.1 mg/kg via oral gavage for 10 consecutive days, 7 days after TBI	7 days post-injury	MWM, WB, TAC, Apoptosis, DHE staining	CCI	Reduced cognitive deficits, ROS generation, and apoptosis after TBI via recovered activation of p38/Nrf2/HO1 signaling pathway	[142]
Silymarin	50 mg/kg via gavage for 20 days before injury	8–10, 11, 18–20, 21 post-injury	Open field, elevated plus-maze, light-dark box, elevated zero-maze, sucrose preference, FST, TST, TNF-α	WD	Decreased anxiety and depression-like behaviours after injury due to reduced TNF-α levels in the prefrontal cortex and hippocampus	[143]
Diosmin	100 mg/kg (p.o.) for 7 days before TBI	–1 h, 1 h, 1,2,15days post-injury	VCS, passive avoidance memory, BBB permeability, brain edema, ELISA	WD	Protective effects against TBI-induced memory and long-term potentiation impairment through reduction of TNF-α concentration in hippocampus	[144]
Catechin	1, 5, 10, 20 or 30 mg/kg daily via gavage up to 28 days post-TBI	3, 5, 7, 14, 21, 28 days pot-injury	Brain infarct volume edema, foot-fault test, MWM, BBB permeability, RT-qPCR, WB	CCI	Catechin prevented tight junction disruption and preserved BBB integrity, reducing post-injury inflammatory reaction	[145]
7,8-dihydroxyflavone	5 mg/kg (i.p) post-TBI either had access to voluntary wheel running for 7 days after injury or were sedentary	7, 14 days post-injury	Barnes maze, voluntary running wheel exercise, WB, rsfMRI	FPI	7,8-DHF enhanced the levels of cell energy metabolism (COII, PGC-1α, AMPK) and hippocampal functional connectivity	[146]
Pycnogenol	50, 100 mg/kg (i.p.) 15 min, 3 h, 6 h post-TBI	2, 5, 7, 12 days post-injury	MWM, FJB, cortical tissue sparing	CCI	No improvement of cognitive ability post-injury in MWM maze task. Pycnogenol suppressed NO through the inhibition of iNOS and also the NF-kB/AP-1 pathway.	[147]
Breviscapine	50 mg/kg (i.v.) post-TBI	1, 4, 7, 14, 21 days post-injury	NSS, RT-PCR, WB, IHC, Immunostaining, TUNEL	WD	Neurobehavioral function improved after treatment due to GSK3β signaling pathway inhibition	[148]
Hydroxysafflor yellow A	10, 30 mg/kg orally post-TBI	6 h, 12 h, 24 h post-TBI	Detection of HSYA, SOD, MDA, CAT, GSH/ GSSG	CCI	HSYA reduced oxidative stress by improving the activities of SOD and CAT, the level of GSH, and the GSH/GSSG ratio. Additionally, it decreased the levels of MDA and GSSG	[149]
Genistein	15 mg/kg (i.p.) 30 min and again 24 h after TBI	–1, 1, 2 days post-TBI	Brain oedema, BBB permeability, ICP, VCS, beam-walk task	WD	Genistein inhibited brain edema, BBB permeability, and improved ICP after TBI. It also improved neurobehavioral performance and motor disorder	[150]
Epicatechin	5, 15, 45 mg/kg by gavage at 3 h after TBI and once daily for 3 days or 15 mg/kg EC at 3 h after TBI and then once daily for 7 days	1, 2, 3, 7, 14, 21, 28 days post-TBI	Neurologic deficit score, forelimb placing test, wire-hanging test, rotarod test, TST, FST, sucrose preference test, IHC, brain water content, Hb, WB, Immunostaining	CCI	EC significantly reduced lesion volume, edema, and cell death and improved neurologic function on days 3 and 28. Cognitive performance and depression-like behaviors were also improved by activating the Nrf2 pathway, inhibiting heme oxygenase-1 protein expression, and reducing iron deposition	[151]
Procyanidins	100 mg/kg (i.v.) PC within 30 min post-TBI	24 h, and 11, 12, 13, 14 days post-injury	MWM, MDA, GSH, SOD, ELISA, WB	CCI	Procyanidins improved cognitive performance by reducing the level of MDA, increasing GSH and activity of SOD, elevating the levels of BDNF, phosphorylation-cAMP-response element-binding protein (pCREB), total CREB, and cyclic AMP (cAMP)	[152]
Proanthocyanidin	Not mentioned	72 h post-injury	Cerebral water content, TBARS, nitrite and nitrate, Thiols	Cold injury	Proanthocyanidin attenuated oxidative and nitrosative stress and decreased brain edema	[153]
(–)-epigallocatechin gallate (EGCG)	0.1% (*w/v*) EGCG pre- and post-TBI. Solution was prepared by dissolving the drug in drinking water	1, 3, and 7 days post-TBI	MWM, IHC, immunostaining for ssDNA and NeuN, lipid peroxidation	CCI	EGCG treatments improved cognitive impairment through inhibiting free radical-mediated neuronal degeneration and apoptotic cell death around the area damaged by TBI.	[154]
Puerarin	200 mg/kg (i.p.) before injury	24 h post-injury	WB, MDA, GSH, Naþ-Kþ-ATPase activity, Myeloperoxidase activity, FJC	WD	Puerarin ameliorated oxidative neurodegeneration after TBI through the activation of PI3K-Akt pathway	[155]
Luteolin	In vivo 10, 30, 50 (i.p.) mg/kg post-TBI In vitro 5, 10, and 25 µM post-TBI	1, 3, 7 days post-TBI	Grip test, brain water content, MDA, GPx activity, TUNEL, IHC, WB, nuclear extraction and electrophoresis mobility shift assay, RT-qPCR, cell viability	WD	Luteolin enhanced the translocation of Nrf2 to the nucleus both in vivo and in vitro, upregulation of heme oxygenase 1 (HO1) and NAD(P)H:quinone oxidoreductase 1 (NQO1). Luteolin neuroprotective effects are possibly mediated by the activation of the Nrf2–ARE pathway	[156]
Naringin	100 mg/kg orally 7 days before and 7 days after the TBI	7 days post-injury	NSS, brain water content, serum and tissue biochemical analysis, WB	WD	Naringin improved behavioral dysfunction by attenuating the increases in MDA and NO; enhancing the activation of SOD; decreasing the over-activation of iNOS; down-regulating the overexpression of IL-1b; and reducing the brain edema.	[157]
Baicalein	30 mg/kg (i.p.) immediately following injury or daily for 4 dayspost-injury	Before injury, 1, 4, 7, 14, 21 days after injury	Rotarod test, y test, mNSS, beam walk test, RT-PCR, WB, IHC, ELISA, FJB	CCI	Baicalein attenuated the contusion’s site expression of TNF-α, IL-1β and IL-6 mRNA and cytokine protein.	[158]

**Table A2:** Summary of preclinical studies on omega-3 fatty acids administration in different models of TBI.

Dosage and Route of Administration	Time Points	Tests and Assays	TBI Model	Main Findings	Ref
DHA: 500 nmol/kg (i.v.) 30 min post-injury	7, 28 days post-injury	mNSS, MRI, ICH	CCI	Reduced lesion size, microglia, and astrocytic reactivity, decreased the accumulation of beta-amyloid precursor protein (APP) at 7 days post-TBI. Reduced the neurofilament light (NFL) levels in plasma at 28 days.	[159]
DHA: 7.5 mg/100 mL (i.p.) 30 min after TBI	1, 3, 7, 14 days post-injury	Brain nitrates and nitrites (NOx), NOR, markers of microglial activation, astrocyte marker, mRNA of inflammation-related genes	CCI	Decreased oxidative stress at day 1 and pro-inflammatory microglial activation at day 3. Decreased oxidative stress, histologic damage, and mRNA markers of microglial pro-inflammatory activation. Improved short term cognitive function.	[160]
NPD 1:50 ng intra-lesionally, immediately following TBI	1,3 days post-injury	FJB, TUNEL, Immuno-staining and lesion size analyses	PBI	NPD1 decreased the lesion area at 72 h	[161]
DHA: 370, 550, 740 mg/kg/day, intragastric administration 30 min after TBI.	1, 4, 7, 14, 21 days post-injury	NSS, MWM TUNEL, Nrf2-ARE pathway-related genes	FPI	Improved neuromotor and cognitive functions, increased anti-apoptotic protein expression, SOD and GPx activity, translocation of Nrf2 to the nucleus. Increased the expression of the downstream factors NAD(P)H:quinone oxidoreductase (NQO-1) and HO-1. Neuroprotective potentially mediated through activating the Nrf2- ARE pathway.	[162]
DHA: 16 mg/kg (i.p) at 30 min, 1, 3, 5 days after TBI	1, 2, 3, 4, 5 days post-injury	NSS, beam walking test and rotarod test, qRT-PCR, WB, TUNEL, IHC	CCI	Improved neurological and cognitive functions. Decreased apoptosis, TLR4 expression, and the expression of inflammatory mediator NF-Kappa B.	[163]
DHA: 370, 740 mg/kg/day (i.v.) 30 min after TBI and daily for 15 days	2, 7 and 15 days post-injury	Beam-walking, MWM, RT-qPCR	FPI	Protection against motor deficits. Inhibition of caspase-3 upregulating the Bcl-2:Bax ratio.	[164]
Resveratrol 50 mg/L via drinking water; Omega3 fatty acids and prebiotics were administered via powdered food (100 g of prebiotic, 300 g of DHA, and 600 g of standard diet per 10 kg of food) for 43 days before TBI.	43–60 days	Neurological test battery, expression of Aqp4, Gfap, Igf1, Nfl, Sirt1, and Tau genes	Focal closed- head	Treatment altered the behavioral performance and prevented injury-related deficits in the longer-term behavior measures, Decreased expression of Aqp4, Gfap, Igf1, Nfl, and Sirt1in the prefrontal cortex.	[165]
DHA: 16 mg/kg (i.p.) at 5 min, 3 to 21 days, after TBI	3, 7, 21 days post-injury	Activation of microglia or macrophages, inflammatory response, neurons expression of the endoplasmic reticulum (ER) stress marker CHOP	CCI	DHA administration reduced neuronal ER stress and subsequent association with microglial or macrophage polarization after TBI. Potential amelioration of TBI-induced cellular pathology	[166]
DHA: 0.1% diet. 1 d before TBI and for 50 days after TBI	1, 2, 3, 12, 28, 41, 47, 50 days post-injury	Inflammatory cytokines, nitrates, and nitrites, MWM, T2-weighted MRI, diffusion tensor imaging, histological exams	CCI	Decreased cognitive impairment, oxidative stress, and white matter injury in adult rats	[167]
ω-3 PUFA: 2 mL/kg (i.p.) 30 min after TBI and daily for 7 days	1, 3, 7 days post-injury	mNSS, brain water content, NISSl staining, microglial activation, immunofluorescent staining, WB, TLR4/NF-κB	Feeney DM TBI model	ω-3 PUFA supplementation inhibited TBI-induced microglial activation and the subsequent inflammatory response by regulating HMGB1 nuclear translocation and secretion and also HMGB1-mediated activation of the TLR4/NF-κB signaling pathway, leading to neuroprotective effects.	[168]
DHA: 16 mg/kg (i.p.) 15 min after the injury and daily for 3 or 7 days.	3, 7 days post-injury.	RT–qPCR, immunoblotting, immunostaining, DTI, MWM	CCI	DHA administration restored hippocampal lysosomal biogenesis and function, demonstrating its therapeutic potential.	[169]
Diet containing 10% corn oil with a fatty acid profile high in ω-6 PUFAs and low in ω-3 PUFAs for 3 days before injury	3, 6, 12 h	Two-photon laser scanning microscopy (2PLSM), parenchymal cell death and reactive oxidative species (ROS), (GSH), neuroprotectin D1 (NPD1)	Closed-head	Neuroprotection by decreasing cell death, ROS formation, and preserving GSH and NPD1 expression.	[170]
DHA (1.2%) enriched diets and/or curcumin (500 ppm) for 2 weeks post-injury	1, 2, 3, 4, 5 days and 2 weeks	Cognitive tests, WB, markers of lipid peroxidation	FPI	DHA alone or in combination improved cognitive functions, decreased oxidative stress and damage to membrane phospholipids. Curcumin complemented the action of DHA on TBI pathology	[171]
DHA = 1.2% of diet coupled with exercise for 12 days	12 days	Expression of acyl-CoA oxidase 1 (Acox1), 17β-hydroxysteroid dehydrogenase type 4 (17β-HSD4), calcium-independent phospholipases A2 (iPLA2), syntaxin-3 (STX-3), and BDNF	FPI	DHA activity was synergistic with exercise. The effects were evident on restoration of membrane homeostasis after TBI, thus supporting synaptic plasticity and cognition.	[172]
Control diet: unhydrogenated soybean oil (70 g/kg). Deficient diet: safflower oil (66.5 g/kg) and soybean oil (3.5 g/kg).	1, 7, 14, 21, and 28 days after TBI	Ccl2, Gfap, and Mmp 9 mRNA levels, MMP-2 and -9 enzymatic activities, lesion volume	CCI	Decreased brain DHA content in TBI rats fed with deficient diet contributed to poorer sensorimotor outcomes after TBI through a mechanism involving modulation of Timp1 expression.	[173]
DHA: 3, 12, 40 mg/kg diet for 30 days prior TBI	1 week after injury	WMW, IHC	WD	Dietary supplementation with DHA increased DHA serum levels, reduced TBI-associated tissue damage (axonal injury counts, markers for cellular injury and apoptosis), and improved memory assessment by the water maze testing.	[174]
